# A Web-Based Lifestyle Intervention Aimed at Improving Cognition in Patients With Cancer Returning to Work in an Outpatient Setting: Protocol for a Randomized Controlled Trial

**DOI:** 10.2196/22670

**Published:** 2021-04-26

**Authors:** A Josephine Drijver, Jaap C Reijneveld, Linda M P Wesselman, Martin Klein

**Affiliations:** 1 Department of Neurology Amsterdam UMC Amsterdam Netherlands; 2 Vrije Universiteit Amsterdam Amsterdam Netherlands; 3 Stichting Epilepsie Instellingen Nederland (SEIN) Heemstede Netherlands; 4 Alzheimer Center Amsterdam Department of Neurology Amsterdam UMC Amsterdam Netherlands; 5 Amsterdam Neuroscience Amsterdam Netherlands; 6 Department of Medical Psychology Amsterdam UMC Amsterdam Netherlands

**Keywords:** cancer, cognitive functioning, lifestyle, web-based intervention, RCT, cancer-related cognitive impairment

## Abstract

**Background:**

A high percentage of patients with cancer experience cognitive impairment after cancer treatment, resulting in a decreased health-related quality of life and difficulty returning to work. Consequently, there is a need for effective treatment options to improve cognitive functioning in these patients. In a healthy aging population, multidomain web-based lifestyle interventions have been found to be effective in preventing cognitive decline and improving cognitive functioning.

**Objective:**

This study aims to investigate the feasibility and effectiveness of the web-based lifestyle intervention Mijn Fitte Brein (My Fit Brain [MFB]) on cognitive functioning in patients with cancer returning to work.

**Methods:**

The study consists of a feasibility study (N=10), followed by a randomized controlled trial (RCT; N=220). Patients will be recruited by their occupational physicians after their return to work following cancer treatment. Mijn Fitte Brein is organized into 4-week cycles in which patients set a lifestyle goal using the Goal Attainment Scale, receive weekly tips and support, and finally evaluate whether they succeeded in achieving this goal. Lifestyle goals are based on 6 domains: physical exercise, diet, sleep, stress, alcohol use, and smoking. In the feasibility study, data on user experience (structured interview) and usability, assessed with the Post-Study System Usability Scale, will be collected and used to optimize Mijn Fitte Brein. In the RCT, patients will be randomized 1:1 between an intervention group and a control group. Patients will be assessed at baseline, 3 months, and 6 months. The primary outcome measure is subjective cognitive functioning, assessed with the Functional Assessment of Cancer Therapy–Cognitive Function (FACT-Cog). Secondary outcome measures are lifestyle, objective cognitive functioning, and work and psychosocial factors.

**Results:**

Recruitment for the feasibility study has started in February 2020. As of July 2020, however, no patients have been enrolled (due to COVID-19 restrictions). The findings of the feasibility study will be used to optimize the Mijn Fitte Brein intervention. Enrollment for the RCT will continue when possible. The feasibility study will take 6 months (including making adjustments to the intervention), and the RCT will take 2 years. The final results are expected in 2024. The results of the feasibility study and the RCT will be published in peer-reviewed journals.

**Conclusions:**

This is the first time the feasibility and efficacy of a multidomain web-based lifestyle intervention will be studied in patients with cancer. If Mijn Fitte Brein is found to be effective in decreasing cognitive complaints in these patients returning to work, it will be a promising treatment option because of being both affordable and accessible.

**Trial Registration:**

Netherlands Trial Register NL8407; https://www.trialregister.nl/trial/8407

**International Registered Report Identifier (IRRID):**

DERR1-10.2196/22670

## Introduction

Cancer and cancer-related treatment can have detrimental effects on cognitive functioning, and complaints of cancer-related cognitive impairment are indeed reported by up to 75% of patients [1]. Besides these cognitive complaints, a subgroup of patients also shows objective cognitive impairment on neuropsychological tests [2]. These findings are of concern, because cognitive impairment negatively affects health-related quality of life (HRQoL) in patients with cancer and survivors of cancer [3]. Not all patients with (mild) cognitive impairment after cancer treatment have cognitive complaints and not all patients who have cognitive complaints show impairment on neuropsychological tests. Therefore, both subjective and objective measures are of interest when studying cognition in this patient group. In clinical practice both outcome measures are useful for patient education and counselling.

In patients with cancer, cognitive impairment affects the ability to work, which also impacts HRQoL [4]. Patients report not being able to keep up with their previous workload and experience a lack of understanding from their environment [5]. Cognitive problems are more frequently reported in patients who experience persistently low work functioning after return to work [6]. Because of these issues, patients are often not able to return to their previous roles or are not able to return to work at all. This highlights the importance of finding treatment options aimed at improving cognitive functioning in patients with cancer returning to work.

Besides the type of antitumor treatment, multiple predictors of cognitive impairment have been identified in patients with cancer [7]. Most of these predictors, such as genetic and sociodemographic factors, are nonmodifiable. Lifestyle, however, is a modifiable predictor and therefore interesting as a target for treatment. Multiple lifestyle factors such as low physical activity, impaired sleep, and unhealthy diet have been identified as risk factors for cognitive decline in patients with cancer [8-11]. Research in healthy individuals and several patient groups has shown that lifestyle interventions are able to improve cognitive functioning or prevent cognitive impairment [12]. In the field of cancer, while several lifestyle interventions have been shown to improve cognitive functioning, most interventions focused on a single lifestyle domain, most frequently targeting physical activity. Yoga, Tai Chi and Qigong, and aerobic exercises have been found to correlate with improved self-perceived cognitive functioning in patients with cancer [13-17]. Research in other populations has shown that interventions targeting multiple lifestyle domains are more effective than single-domain interventions. For example, the Finnish Geriatric Intervention Study to Prevent Cognitive Impairment and Disability (FINGER) trial, which focused on diet, exercise, cognitive training, and vascular risk monitoring [18], was found to be effective in improving or maintaining cognitive function in an at-risk, but otherwise healthy, elderly population [19].

One disadvantage of these face-to-face multidomain lifestyle interventions such as the FINGER trial is that multiple weekly visits to the training and counseling facility are required [20]. Besides the burden of multiple visits, the costs of providing/offering these interventions are considerable. More affordable alternatives are web-based interventions, which have been studied in diverse populations [21]. Such interventions are not only more affordable, but are also easily accessible for patients, as currently 97% of the Dutch population has internet access [22]. Recently the Healthy Ageing Through Internet Counselling in the Elderly (HATICE) trial studied the effect of an internet-supported intervention aimed at decreasing cardiovascular risk factors and the risk of cognitive decline in an older population [23]. The first results showed that this web-based intervention was feasible and effective [24].

In patients with cancer, no web-based multidomain lifestyle interventions aimed at improving cognitive functioning have been studied so far. This protocol describes a feasibility study and a subsequent randomized controlled trial (RCT) aimed at evaluating the feasibility and effectiveness of such an intervention in patients with cancer returning to work. In a self-motivated intervention, patients do not receive guidance from a counselor and are expected to work through the program independently. The study aims to establish the feasibility of a self-motivated lifestyle intervention to this population, and whether the program is beneficial for the cognitive functioning of patients. In addition, the underlying factors mediating effectivity will be studied.

## Methods

### Approval

The study protocol has been approved by the Medical Ethical Committee of the VU Medical Center. The study will be conducted according to the principles of the Declaration of Helsinki, and in accordance with the Medical Research Involving Human Subjects Act (WMO). The trial has been registered at the Netherlands Trial Registry (NL8407).

### Design

#### Baseline Assessment

**Figure 1:**
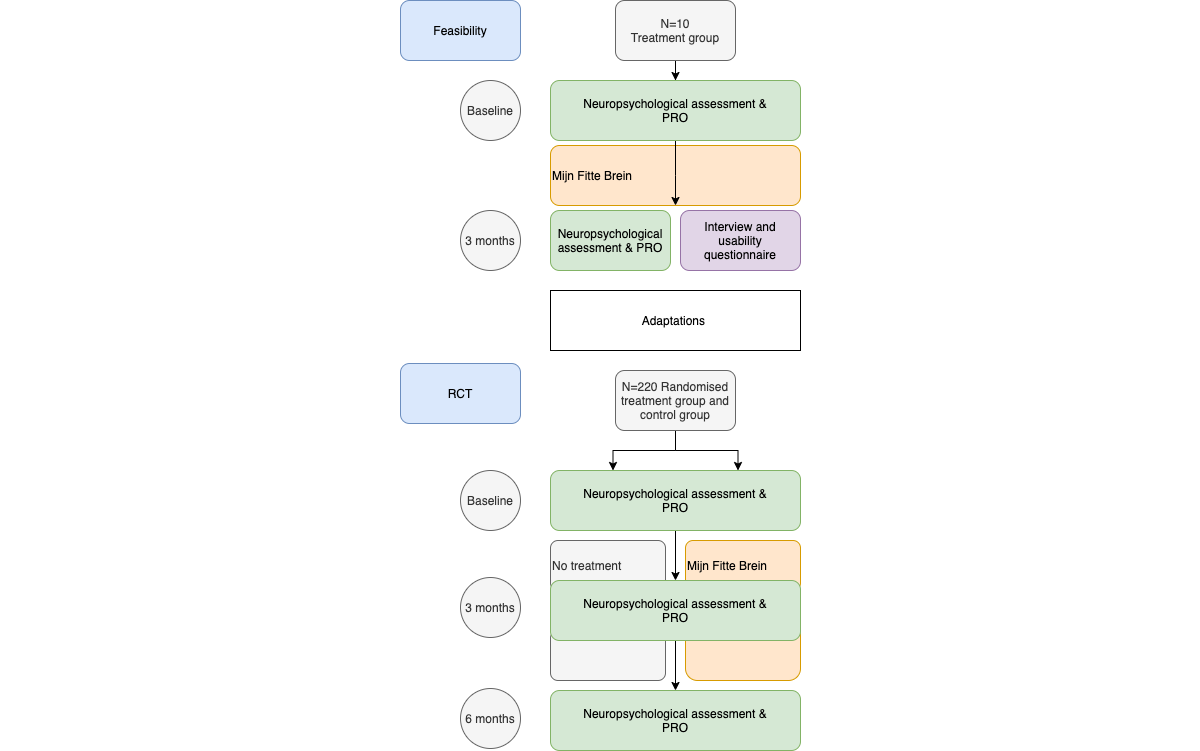
Design of the feasibility study and randomized controlled trial. PRO: Patient Reported Outcomes.

This study consists of a feasibility study and an RCT (Figure 1) in an outpatient setting. Participants will be informed of the study by their physician at a Dutch occupational health institute.

Baseline assessment will take place at the hospital or at the participant’s home, depending on the participant’s preference. A member of the research team will answer any of the participant’s questions before informed consent is given. The researcher, who has been trained on the test protocol, administers a neuropsychological test battery and the participant fills in the questionnaires (see the “Outcome Measures” section) online. The first visit will take approximately 2 hours. Follow-up assessments will adhere to the same protocol.

The information on the RCT protocol is provided according to the CONSORT 2010 checklist (Multimedia Appendix 1) [25].

#### Feasibility Study

In the feasibility study, 10 participants will get access to the Mijn Fitte Brein (My Fit Brain [MFB]) intervention for 3 months.

After the baseline assessment the participants will receive an email with login information to access the web-based Mijn Fitte Brein intervention for 3 months. The participants are instructed to use Mijn Fitte Brein weekly during this period. At the 3-month follow-up, the cognitive assessment will be repeated and the participants will fill in the questionnaires. For this feasibility study specifically, the researcher will interview the participants about their experience with Mijn Fitte Brein. In addition, the participants will fill out a questionnaire about the usability of the platform.

#### Randomized Controlled Trial

In the RCT, 220 participants will be randomly assigned to the intervention group or the control group.

The RCT uses a parallel design. Randomization will be performed by a researcher not associated with the testing of participants, in Castor EDC [26]. Variable block randomization will be used with blocks of 8, 10, and 12, with each condition (Mijn Fitte Brein/No treatment) randomly generated 50% within each block to ensure an unpredictable allocation sequence with equal numbers of participants in each group at the completion of each block. The allocation ratio is 1:1. Blinding of patients is not possible due to the nature of the intervention. Assessors of the neuropsychological examination will be blinded.

Participants in the treatment group will get a 6-month access to Mijn Fitte Brein, whereas those in the control group will get access after a 6-month waiting period. Participants will be assessed at baseline, 3 months, and 6 months. The participants will not be interviewed at the end of the study. Control group participants are allowed to find other help sources for their cognitive problems, and this is recorded in the study. However, they are not allowed to participate in other studies specifically aimed at improving cognition.

### Participants

#### Eligibility Criteria

To be eligible to participate in this study, potential participants must meet all of the following criteria: be over 17 years of age, be employed, have received primary treatment (chemotherapy, immunotherapy) for a recent noncentral nervous system cancer, completed treatment (except for hormonal treatment), report cognitive complaints, and provide written informed consent.

Exclusion criteria are primary or secondary central nervous system tumors, insufficient mastery of the Dutch language, self-reported insufficient computer skills, history of brain injury with loss of consciousness, history of brain surgery, currently under active treatment for psychiatric or neurodegenerative disorders, self-reported substance abuse, and severe visual impairments.

#### Recruitment

All participants will be informed of the study by their occupational health physician. If interested, participants will be approached by one of the researchers and receive further information. When inclusion criteria are met, the researchers will schedule the first visit. During this visit, all participants will provide written informed consent before the start of the study activities.

#### Sample Size

In the feasibility study 10 participants will be included. Regarding the subsequent RCT, thus far, no comparable studies have been published using a similar intervention in a population of cancer survivors. Therefore, sample size is calculated based on the minimal clinically important difference (MCID) on the Functional Assessment of Cancer Therapy–Cognitive Function (FACT-Cog; a questionnaire to measure subjective cognitive functioning in patients with cancer and the primary outcome of the RCT). The MCID was found to be 9.6 points when using an anchor-based approach [27]. In the same population, an SD of 23.3 was found. Using a significance level of α=.05 and test power (1–β) of 80% leads to a sample size of 188 participants equally divided into 2 groups. Assuming an estimated drop-out rate of 15%, a total of 220 patients will be included in the RCT.

### Intervention: Mijn Fitte Brein

#### Description

Mijn Fitte Brein (translation: *My Fit Brain*) is a web-based lifestyle intervention based on the Brain Aging Monitor (BAM) [28]. Use of the BAM was associated with significant improvement in cognition-associated lifestyle factors in healthy older adults (40-60 years) [29]. The BAM has been adapted where necessary to conform to current lifestyle guidelines, to be suitable for the patients in this study, and to comply with the EU and European Economic Area General Data Protection Regulation (GDPR). Mijn Fitte Brein is built on the Minddistrict platform [30].

**Figure 2:**
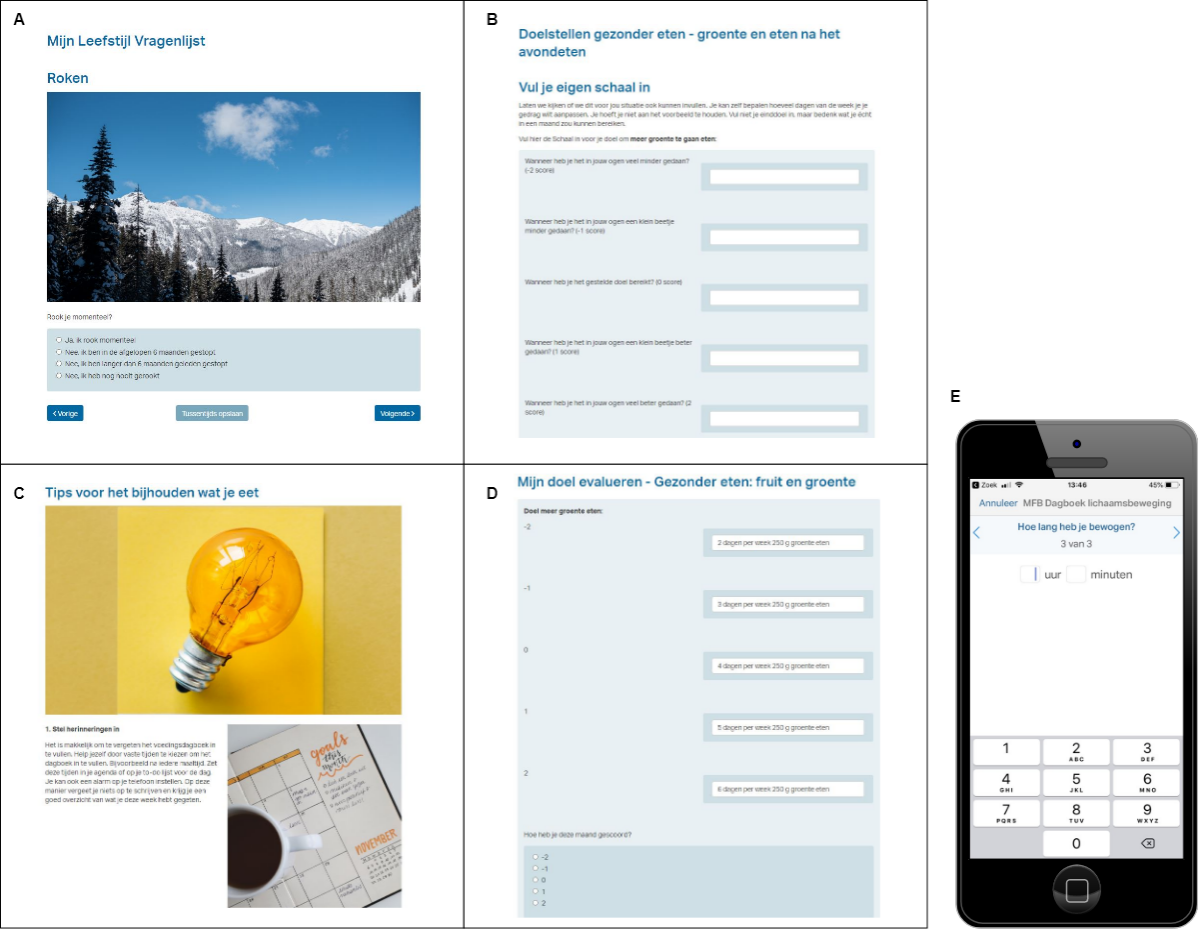
Screenshots showing the four steps of the MFB intervention. A: lifestyle questionnaire, B: goal setting, C: tips, D: goal evaluation, E: diary on the smartphone.

The Mijn Fitte Brein web-based intervention is constructed around 6 lifestyle domains: physical exercise, nutrition, sleep, stress, alcohol, and smoking. It consists of a lifestyle questionnaire followed by 3 modules (Figure 2): a goal setting module, an information and diaries module, and a goal evaluation module. See Multimedia Appendix 2 for the monthly schedule of Mijn Fitte Brein.

##### Questionnaire and Feedback

The first time a user logs onto the website, a questionnaire covering all 6 lifestyle domains is presented. Subsequently, the user receives feedback tailored to the provided answers. If national guidelines for the specific domains are available, the feedback states whether the user adheres to these guidelines. Otherwise, feedback includes a comparison of the user’s current behavior with the national average. It also contains general recommendations on how to improve lifestyle in the specific domains. Feedback concludes with recommendations about the domains where the participant could improve most. Finally, the user is asked to choose a domain to work with, and to set a goal for the upcoming month. See Table 1 for possible goals within each lifestyle domain.

**Table 1:** Domains and goals.

Domain	Goals
Physical exercise	Start moving, start exercising, increase exercising
Nutrition	Healthy eating^a^
Smoking	Quit smoking, decrease smoking
Alcohol	Decrease daily alcohol consumption, decrease weekly alcohol consumption, decrease glasses of alcohol when drinking
Stress	Reduce stress
Sleep	Improve sleep quality, increase sleep duration, improve sleep pattern

^a^For the goal healthy eating users can select 2 subgoals out of the following: “have breakfast more often,” “eat more vegetables,” “eat more fruit,” “eat more fish,” “eat less unhealthy snacks,” and “eat less often after dinner.”

##### Goal Setting

Next, the user is referred to the goal-setting module. First, the user receives an explanation of the goal-setting method that is used in Mijn Fitte Brein: Goal Attainment Scale (GAS) [31]. Second, following the GAS method, the user has to complete a scale ranging from –2 (I have made minimal progress) to 2 (I have done a lot better than my original goal). The user specifies his/her goal by describing a situation for each level on the scale.

For each potential goal, Mijn Fitte Brein provides instructions and recommendations that assist the user with realistic goal setting. To further aid goal setting, an example GAS and case description, as well as step-by-step instructions are presented. After the user has set a goal, he/she receives reinforcing feedback aimed at initiating behavior change. Last, the user specifies the goal by completing a personal GAS. For an example of the goal-setting module, see Multimedia Appendix 3.

##### Information and Diaries

The day after completing the goal-setting module, the user is given access to the first part of the training on the Minddistrict dashboard. Every week a new training module becomes available, providing tips and tricks tailored to the specific goal the user has chosen. For example, tips on how to overcome barriers in reaching your goal, mindfulness exercises, and training schedules for physical activity are shared. The user receives an email alert when a new part of the training becomes available.

In addition, daily diaries are made available for the user to track his/her progress. The user can access the diary on both a smartphone and a computer. The diaries are tailored to each goal; for example, in the physical activity diary, users can track which activity they performed and how much time they spent exercising. Similarly, in the sleep diary, users can track their hours of sleep and factors that might have influenced their sleep quality. In the diary, graphs are made available so that users can keep track of their progress. A notification on their phone prompts users to complete their diaries. The diary is also visible on the dashboard when users log-on to the Minddistrict platform.

In the second and third week of every month, the user evaluates the previous week and is encouraged to identify factors that hindered or facilitated his/her progress.

##### Goal Evaluation

**Figure 3:**
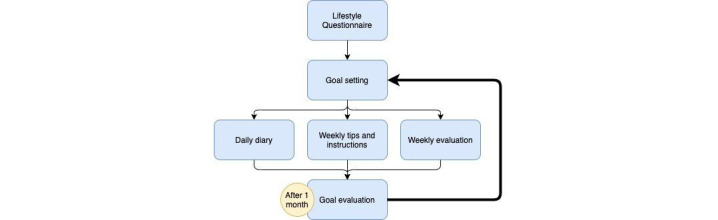
Cycle of the MFB intervention.

After 4 weeks, the user revisits the previously created GAS. Using this scale, the user indicates whether and to what extent the goal was reached. This evaluation is followed by personalized feedback. If a goal has been met, the user is asked to set a new goal. If the user has failed to reach a goal, he/she receives tips on how to set a realistic goal for the next month; they can also choose the same goal. Figure 3 shows the cycle of Mijn Fitte Brein that repeats each month.

##### Behavior Change Techniques

Mijn Fitte Brein facilitates behavior change and stimulates adherence. Behavior change is enabled by multiple methods integrated in the platform. The use of GAS in the goal-setting module helps patients to set realistic and attainable goals [31]. An overview of all integrated behavior change techniques according to the Behavior Change Technique (BCT) taxonomy [32] can be found in Multimedia Appendix 4.

#### Technical Support

The intervention is completely web based and self-applied, meaning that no involvement of health care professionals is needed for participants to use the program. For questions regarding the use of Mijn Fitte Brein, patients can contact the research team. For technical help users can contact the Minddistrict helpdesk.

#### Data Logging

The Minddistrict platform automatically stores data such as the number of logins, when a module is started and finished, when a diary is completed, and the total time spent on the platform.

### Outcome Measures

#### Feasibility Study

##### Primary Outcome Measure: Identification of Areas of Improvement

The patients in the feasibility study will be interviewed after the intervention period. Using semistructured interviews, information about the usability and the patients’ experience of Mijn Fitte Brein will be collected. The interview will cover the following themes: the intervention, lifestyle domains, information on cognition and lifestyle, barriers and facilitators for using the intervention, and whether Mijn Fitte Brein facilitated behavior change. In addition, patients will be asked how they think the intervention could be further improved for patients with cancer. For the interview guideline, see Multimedia Appendix 5.

This feedback will be used to adjust Mijn Fitte Brein for the RCT. The interview will be recorded and summarized. Data will be organized by 2 researchers (AD and MK) to identify areas of improvement. Summaries will be used to gain insight into the user experience of the patients. When patients report difficulty with the use of the intervention, changes will be made to the platform accordingly. When an area of improvement has been suggested more than once and addition to the online intervention is feasible, the platform will be adjusted accordingly before the start of the RCT.

Findings in the log data will also be used to identify changes in adherence and usage of Mijn Fitte Brein. Participants are also asked to clarify reasons for their usage and adherence. These explanations will be used to improve the platform and thereby optimize adherence in the future.

##### Other Outcome Measures

###### Usability

The usability of Mijn Fitte Brein will be reported using the Post-Study System Usability Scale (PSSUQ) [33]. The PSSUQ questionnaire consists of 16 items on usability (scores ranging from 1 to 7). The total score ranges from 16 to 112, with higher scores indicating better usability ratings.

###### Log Data

Log data are used to study the use of Mijn Fitte Brein and adherence.

The use of the platform will be described by the number of logins and the time spent on Mijn Fitte Brein. Furthermore, the number of completed modules, goals, and diaries will be counted and used to further specify the use of Mijn Fitte Brein.

Adherence will be described by reporting the average number of completed sessions, and the number of completed active (diaries/exercised) versus passive (information) sessions. Finally the number of people that never logged in will be reported.

##### Randomized Clinical Trial

###### Primary Outcome Measure: Subjective Cognitive Functioning

Subjective cognitive functioning is the primary outcome measure of the RCT. The Dutch version of the FACT-Cog questionnaire [34] will be used to indicate self-perceived cognitive impairment. The questionnaire has high internal consistency and satisfactory test–retest reliability [35]. The FACT-Cog consists of 37 items related to cognitive problems due to treatment in patients with cancer. The items are divided into 4 categories: perceived cognitive impairments (20 questions), comments from others (4 questions), perceived cognitive abilities (9 questions), and impact on HRQoL (4 questions). All items are rated on a 5-point Likert scale ranging from 0 (“Never” or “Not at all”) to 4 (“Several times a day” or “Very much”). The total score for the FACT-Cog ranges from 0 to 148, with a higher score indicating better perceived cognitive functioning. The threshold for MCID was set at 9.6 points [27].

###### Other Outcome Measures

####### Objective Cognitive Functioning

A neuropsychological test battery is used to measure functioning of multiple cognitive domains, taking approximately 1.5 hours to complete. This test battery is based on the core battery–recommended tests by the International Cognition and Cancer Task Force [36]. These tests are specifically selected for their ability to gauge a broad range of neurocognitive functions in patients with cancer. Additional tests based on previous research and our clinical experience were added to this core battery [37]. The battery consists of widely used standardized psychometric instruments that have been shown to be sensitive to the negative effects on cognition of cancer treatment in other clinical trials. For an overview of the tests, see Table 2. The neuropsychological test data will be scored according to standard scoring algorithms. The included tests have published normative data that take into account age and, where appropriate, education, gender, and handedness. The test results for the patients can also be expressed in a standard form denoting their relation to control data with the use of a z-transformation: z = (x-m)/SD, where x is the observed score for the patient; m is the average score for healthy controls; and SD is the standard deviation for healthy controls. This provides measures of functioning which are comparable across tests.

**Table 2:** Overview of neuropsychological test battery.

Test	Cognitive domain
Hopkins Verbal Learning Test–Revised [38]	Verbal memory
Trail Making Test A + B [39]	Flexibility
WAIS-III Digit Symbol Substitution Test [40]	Information processing/attention
Eriksen Flanker Task [41]	Response inhibition
Rey Complex Figure [42]	Visuospatial construction and recall
WAIS-III Digit Span Backwards [40]	Working memory
Stroop Color Word Test [43]	Attention, response inhibition
Tower of London [44]	Planning/executive functioning
Controlled Oral Word Association Test [45]	Verbal fluency
Behavioural Assessment of the Dysexecutive Syndrome (BADS) 6 Elements Test [46]	Planning/executive functioning

####### Health-Related Quality of Life

The EORTC Core Quality of Life Questionnaire (EORTC QLQ-C30) will be used to establish the patients’ HRQoL [47]. This questionnaire is widely used in clinical trials to evaluate HRQoL in patients with cancer. The questionnaire consists of 30 items, which are distributed over 5 functional scales (physical, role, cognitive, emotional, and social), 3 symptom scales (fatigue, pain, and nausea and vomiting), a global HRQoL scale, and additional items to address other common symptoms. The patient is asked to indicate which number on a 4-point Likert scale applies best to them. The total global health status scores range from 0 to 100, with a high score indicating high HRQoL.

####### Psychosocial Factors

Self-efficacy will be measured using the Dutch General Self-Efficacy Scale [48]. This scale consists of 10 items that measure the beliefs that one’s actions are responsible for successful outcomes. Items are scored on a 4-point scale, and the total score ranges from 10 to 40, with a higher score indicating more self-efficacy.

To measure symptoms of fatigue, the *Checklist Individual Strength* (CIS) will be used [49]. The CIS is widely used for studying fatigue in a working population. This questionnaire consists of 20 items divided over 4 scales: subjective experience of fatigue, concentration, motivation, and physical activity. Items are scored on a 7-point Likert scale. The total score ranges from 20 to 140, with a higher score indicating more fatigue-related problems.

Anxiety and depression state will be measured using the Hospital Anxiety and Depression Scale (HADS) [50]. The scale is widely used to screen for emotional distress in both clinical and research settings. The HADS consists of 2 subscales (each with 7 items). Each item is rated on a 4-point scale from 0 to 3, yielding a maximum score of 21 for each subscale. A higher score indicates more symptoms of anxiety or depression.

One item of the work ability index [51], “current work ability compared with the lifetime best,” will be used to assess total ability to work. This item is suitable to be used as a proxy for the full work ability index [52]. The item has a score between 0 (completely unable to work) and 10 (work ability at its best).

To measure cognitive functioning specific for the work context, the Dutch version of the Cognitive Symptom Checklist–Work [53] will be used. This questionnaire consists of 19 items measuring work-specific cognitive problems in patients with cancer. Items are rated on a 5-point scale ranging from 0 (never) to 4 (always). Total scores range between 0 and 100, with a higher score indicating more cognitive symptoms.

To measure work functioning, 2 scales of the Dutch version of the Work Role Functioning Questionnaire will be used [54]. Both the mental and social demands scale, consisting of 7 items, and the flexibility demands scale, consisting of 5 items, will be included. Subscale scores are scored by taking the sum score of the items, divided by the number of items, and then multiplied with 25 to obtain a score between 0 and 100, with higher scores indicating better work functioning.

####### Lifestyle Factors

The lifestyle questionnaires are used as input for goal setting in the Mijn Fitte Brein intervention. There are separate questionnaires for the following domains: alcohol consumption status, physical activity, exercise, healthy nutritional behavior, smoking status, sleep status, and stress status. The questionnaires are based on the recommendations for physical exercise, nutrition, and alcohol use by the Health Council of the Netherlands [55,56]. For a detailed description of the questionnaires, see Multimedia Appendix 6.

###### Data Management and Security

The Minddistrict platform is hosted by Intermax. Intermax is SO27001 and NEN-7510 certified. Minddistrict has received an EU Declaration of Conformity (No. 2017/590-01). Data are collected and stored according to the GDPR. All participants are assigned an identification number that is used for the collection of the raw data and in the data analysis.

Data are stored online on the European server of Castor EDC. Paper and pencil tests and informed consent files will be stored separately in a locked file cabinet at Amsterdam UMC, location VUmc, Amsterdam, The Netherlands. Upon completion, questionnaire data will be directly recorded into an online system (Castor EDC). The data will then be transferred into a statistical database for analysis. Raw data will be stored for 15 years.

An on-site monitor has been assigned to the study to make sure the right procedures are followed and the correct documentation is provided before, during, and after the study.

###### Data Analysis

The data analysis plan has been developed in collaboration with a statistician experienced in statistics for clinical cancer research.

####### Feasibility Study

For adherence and use of the intervention, descriptive statistics (eg, percentages, median, and interquartile range) of log data and the PSSUQ will be generated.

Qualitative data collected during the interviews will be summarized and organized by 2 researchers (AD and MK). Data will be categorized by theme to create an overview of possible areas of improvement.

####### Randomized Controlled Trial

######## Descriptive Statistics and Demographics

Descriptive statistics (means [SD]) will be generated for the range of outcome measures and demographics (age, gender, level of education, cancer type, time since diagnosis, time since treatment completion, treatment type, scores on all questionnaires, and scores on neuropsychological tests).

Demographics of the interventions and control group will be compared, including level of education, fatigue (MFI-20 scores), anxiety and depression (HADS scores), using a chi-square test or independent samples *t* tests where appropriate.

######## Effect of Mijn Fitte Brein on Cognitive Complaints Over Time

To determine the effects of between-subjects factor intervention, within-subjects factor time points (baseline, 3-month follow-up, and 6-month follow-up), and their interaction on cognitive complaints and secondary study parameters, linear mixed models will be used. In case of a significant intervention × timepoint interaction, effect sizes after 3 and 6 months (or a change from baseline) will be computed with an independent samples *t* test. The assumption of normality will be checked using histograms. In case of violations, nonparametric procedures will be used. Two-tailed probability tests will be used in all inferential statistical testing. A result will be considered significant at *P*≤.05. A change equal to or greater than the MCID (9.6 points) will be considered as a meaningful change on the FACT-Cog.

######## Moderating Effects

To study the moderating influence of other factors on the effect of Mijn Fitte Brein, a linear mixed model will be used, including between-subjects factors intervention and moderator, and within-subject factor time, all 2-way interactions, and the 3-way interaction. The following possible moderators will be tested: age, gender, self-efficacy (DGSES), cancer type, type of treatment, work performance (Work Role Functioning Questionnaire), level of education, mood (HADS), and fatigue (CIS). Bonferroni-adjusted α levels will be used to control for type I errors due to multiple comparisons.

Statistical analyses will be conducted using SPSS 22 (IBM).

####### Bias Due to Loss to Follow-Up and Missing Data

In both the feasibility study and the RCT, participants who do not complete the study will be questioned about their reasons for dropping out. The responses will be logged and reported.

In the RCT, the number of participants lost to follow-up and their reasons will be compared between groups. In addition, the characteristics of study completers will be compared with the characteristics of noncompleters. Finally, we will analyze missing data to determine whether these are missing at random or not. In the data analysis a linear mixed model will be used, which is able to compensate for missing data.

## Results

The feasibility study started in February 2020. Because of government regulations for COVID-19, patient recruitment has been deferred for the foreseeable future. As of July 2020, no patients have been enrolled. The findings of the feasibility study will be used to optimize the Mijn Fitte Brein intervention. Enrollment for the RCT will continue when possible. The feasibility study will take 6 months (including making adjustments to the intervention), and the RCT will take 2 years. The final results are expected in 2024. The results of the feasibility study and the RCT will be published in peer-reviewed journals.

Apart from sharing the outcomes of this study with the scientific community, we will approach health insurance companies to make Mijn Fitte Brein more broadly available when it is found to be effective in improving cognition in patients with cancer. The results of the study will also be shared via media such as (local) newspapers and social media.

## Discussion

This protocol presents a feasibility study and an RCT to investigate whether a multidomain web-based lifestyle intervention, Mijn Fitte Brein, is feasible and effective in a population of patients with cancer returning to work. Cancer-related cognitive impairment is reported in up to 75% of patients with cancer and has a high impact on HRQoL. Lifestyle factors are associated with cognitive functioning in both healthy and cancer populations. The modifiability of lifestyle makes it a suitable target for interventions aimed at improving cognitive functioning in patients with cancer.

By providing patients with Mijn Fitte Brein during the time they are returning to work, the negative impact of cognitive impairment on work performance and experience might be decreased. Furthermore, improvement in lifestyle might benefit (psychological) health and improve HRQoL. Another strength of the study is the 2-phase study design consisting of a feasibility study and an RCT. The findings of the feasibility study will be used to further improve and adjust the intervention for the RCT, resulting in an intervention tailored to the wishes of the patient population. The use of an RCT design makes it possible to control for naturally occurring improvement of cognitive functioning over time and learning effects on the neuropsychological assessment. The assessments at baseline, 3 months, and 6 months make it possible to follow the trajectory of changes in both lifestyle and cognitive functioning.

The study has some possible limitations. First, because of the nature of the study, it is not possible to blind patients to the condition they are assigned to. This might influence test performances. To limit effects of observer bias, test personnel will be blinded to the group the participant is assigned to. Second, there is a chance patients assigned to the control group will have a higher drop-out rate due to not receiving immediate access to the intervention. To prevent this, the control group will be given access to the intervention after the study period. Finally, because the patients are selected by their treating occupational physician, there is a risk of selection bias. However, the occupational physicians are instructed by the researchers to minimize this bias.

This study is the first to investigate the use of a multidomain web-based lifestyle intervention in a cancer population with the goal to improve cognitive functioning. If Mijn Fitte Brein is found to be feasible for our study population and effective in decreasing cognitive complaints in patients with cancer returning to work, it might be implemented in clinical practice. Being both affordable and accessible, Mijn Fitte Brein could contribute to better cognitive functioning and thereby increase the return-to-work rate and quality of life in patients with cancer.

## Appendix

Multimedia Appendix 1 CONSORT 2010 checklist of information to include when reporting a randomized trial.

Multimedia Appendix 2 Monthly schedule of MFB.

Multimedia Appendix 3 Example of the goal setting module.

Multimedia Appendix 4 Overview of the behavior change techniques used, according to the BCT taxonomy.

Multimedia Appendix 5 Topic list for the semi-structured interviews for the feasibility study.

Multimedia Appendix 6 Description of the lifestyle questionnaires in MFB.

## Abbreviations

BAM: Brain Aging Monitor
GAS: Goal Attainment Scale
GDPR: General Data Protection Regulation
HRQoL: health-related quality of life
MCID: minimal clinically important difference
PSSUQ: Post-Study System Usability Questionnaire
RCT: randomized controlled trial
