# Supplementary material for: A Web-Based Lifestyle Intervention Aimed at Improving Cognition in Patients With Cancer Returning to Work in an Outpatient Setting: Protocol for a Randomized Controlled Trial
Source: JMIR Res Protoc. 2021 Apr 26;10(4):e22670. doi: 10.2196/22670 (PMC8111506; doi:10.2196/22670)
Supplement: Multimedia Appendix 2 [file resprot_v10i4e22670_app2.docx]

##
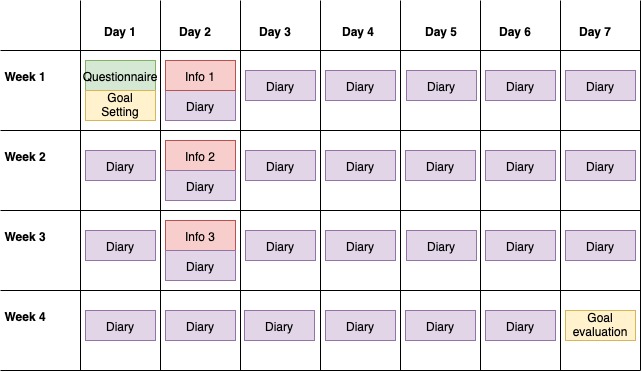


*Questionnaire:* the lifestyle questionnaire.

*Goal Setting*: choosing a domain and setting a goal using the Goal Attainment Scaling method.

*Info 1/2/3*: information and tips of the chosen goal. *Diary*: daily diary, depending on the chosen goal.

*Goal Evaluation*: Evaluation of the GAS goal set at the beginning of the month.
