# Supplementary material for: A Web-Based Lifestyle Intervention Aimed at Improving Cognition in Patients With Cancer Returning to Work in an Outpatient Setting: Protocol for a Randomized Controlled Trial
Source: JMIR Res Protoc. 2021 Apr 26;10(4):e22670. doi: 10.2196/22670 (PMC8111506; doi:10.2196/22670)
Supplement: Multimedia Appendix 3 [file resprot_v10i4e22670_app3.docx]

First, the participant is shown an explanation of how the goal setting module works:


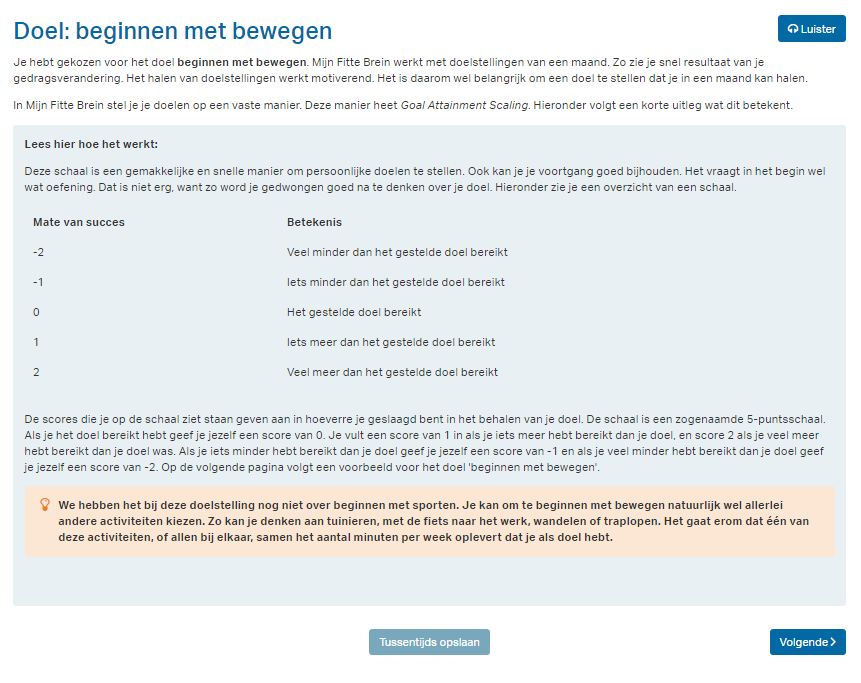


Translation:

Goal: start moving

You have chosen the goal **start moving.** My Fit Brain works with monthly goals, so you will notice results quickly. Reaching you goals is motivating. It is important to set goals that you are able to reach within a month.

My Fit Brain uses a fixed method for setting goals. This method is called Goal Attainment Scaling. Below you will read a short explanation of what this entails.

**Read how it works:**

This scale is a quick and easy way to set personal goals. It also helps you to keep track of your goals. In the beginning, some practice is needed. That’s okay, because it forces you to think about your goal. See an overview of the scale below.

| **Level of success** | **Meaning** |
| --- | --- |
| -2 | Did a lot less than the set goal. |
| -1 | Did a bit less than the set goal |
| 0 | Reached the set goal |
| 1 | Did a bit better that the set goal |
| 2 | Did much better than the set goal |

The scores on the scale represent how well you have performed in reaching your goal. The scale is a so-called 5-point scale. If you have reached your goal, you give yourself a score of 0. If you did a bit better than you set out to do in your goal, your score is 1. If you did much better than your original goal, you get a score of 2. If you did a bit less than you planned in your goal, your score is -1. If you did much less than you planned to, your score is -2. On the next page you will see an example for the goal: “start moving”.


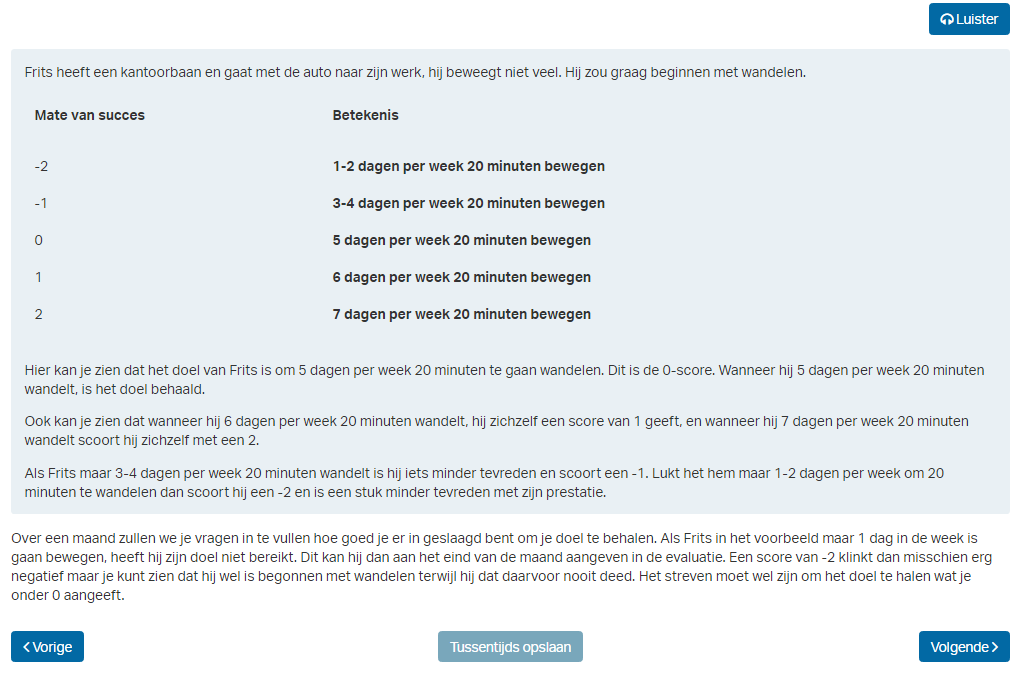


Frits has an office job and travels to work by car, he doesn’t move often. He would like to start going on walks. This is how he completed the scale.

| Level of success | Meaning |
| --- | --- |
| -2 | Walk 1-2 days a week for 20 minutes |
| -1 | Walk 3-4 days a week for 20 minutes |
| 0 | Walk 5 days a week for 20 minutes |
| 1 | Walk 6 days a week for 20 minutes |
| 2 | Walk 7 days a week for 20 minutes |

You can see that it is Frits’ goal to walk 5 days a week for 20 minutes. This is the 0 score. If he manages to walk 20 minutes for 5 days he has reached his goal.

If he manages to walk 6 days, his score is 1, and if he goes on a walk for 7 days, his score is 2.

If Frits only goes on a 20 minute walk for 3 or 4 days, he is less satisfied and his score is -1. If he only goes on a walk for 1 or 2 days, he scores -2.

In a month you’ll be asked whether you have managed to reach your goal. If Frits in the example has just started to walk 1 day a week, he hasn’t reached his goal. In the end of the month he can indicate this at the evaluation. A -2 score might sound very negative, but he still managed to start walking when he wasn’t walking before. Nonetheless, you should still aim to reach the goal you identify under 1.


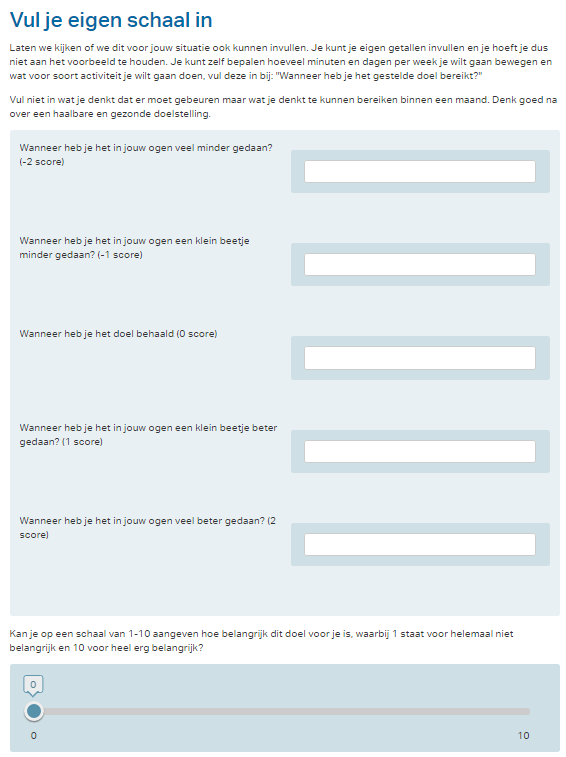


Let’s see if you can complete the scale according to your situation. You can choose your own activity and numbers, so you don’t have to copy the example. Decide how many minutes you want to move and what type of activity you want to do. Put this in the field at: “When have you reached your goal?”.

Don’t fill in what you think you *should* do, but what you think you actually *can* *accomplish* within a month. Consider an attainable and healthy goal.

| When have you done a lot less? (-2) |  |
| --- | --- |
| When have you done a bit less? (-1) |  |
| When have you reached your goal? (0) |  |
| When have you done a bit better? (1) |  |
| When have you done a lot better? (2) |  |

Can you indicate the importance of this goal, from 0: = not important at all, to 10: very important?
