# Supplementary material for: A Web-Based Lifestyle Intervention Aimed at Improving Cognition in Patients With Cancer Returning to Work in an Outpatient Setting: Protocol for a Randomized Controlled Trial
Source: JMIR Res Protoc. 2021 Apr 26;10(4):e22670. doi: 10.2196/22670 (PMC8111506; doi:10.2196/22670)
Supplement: Multimedia Appendix 4 [file resprot_v10i4e22670_app4.pdf]

Behavior change techniques	Example of the BCT in MFB
**1. Goals and planning**	
1.1 Goal setting (behavior)	A monthly goal setting module
1.2 Problem solving	Exercises to identify barriers for the desired behavior
1.4 Action planning	Specific times and dates are set for the desired behavior
1.5 Review behavior goals	Goal progression is evaluated weekly
1.6 Discrepancy between current behavior and goal	n.a.
1.7 Review outcome goal(s)	The outcome goal is reviewed monthly
1.8 Behavioral contract	Social contract is available in for example the quitting smoking goal
1.9 Commitment	n.a.
**2. Feedback and monitoring**	
2.1 Monitoring of behavior by others without feedback	n.a.
2.2 Feedback on behavior	Diary input is presented in graphs to monitor frequency and quantity of behavior
2.3 Self-monitoring of behavior	The daily diaries facilitate self-monitoring of behavior
2.4 Self-monitoring of outcome(s) of behavior	The weekly evaluation prompts users to evaluate the effect of their behavior changes
2.5 Monitoring of outcome(s) of behavior without feedback	n.a.
2.6 Biofeedback	n.a.
2.7 Feedback on outcome(s) of behavior	n.a.
**3. Social support**	
3.1 Social support (unspecified)	Finding social support is advised
3.2 Social support (practical)	n.a.
3.3 Social support (emotional)	n.a.
**4. Shaping knowledge**	
4.1 Instruction on how to perform a behavior	Training schedules are provided for exercise goals, relaxation practices etc.
4.2 Information about antecedents	Advise to think about situations and factors that facilitate the unwanted behavior
4.3 Re-attribution	n.a.
4.4 Behavioral experiments	n.a.
**5. Natural consequences**	
5.1 information about health consequences	Effects of behavior on (brain) health are mentioned
5.2 Salience of consequences	n.a.
5.3 Information about social and environmental consequences	n.a.
5.4 Monitoring of emotional consequences	The daily diaries contain space for reporting feelings
5.5 Anticipated regret	n.a.

5.6 Information about emotional consequences	n.a.
**6. Comparison of behavior**	
6.1 Demonstration of the behavior	n.a.
6.2 Social comparison	Scores on the questionnaire are compared to the national average when available
6.3 Information about others' approval	n.a.
**7. Associations**	
7.1 Prompts/Cues	Users receive reminders when a new module or diary is ready
7.2 Cue signaling reward	n.a.
7.3 Reduce prompts/cues	n.a.
7.4 Remove aversive stimulus	Smokers are advised to remove all objects that are associated to smoking
7.5 Remove aversive stimulus	n.a.
7.6 Satiation	n.a.
7.7 Exposure	n.a.
7.8 Associative learning	n.a.
**8. Repetition and substitution**	
8.1 Behavioral practice/rehearsal	n.a.
8.2 Behavioral substitution	Suggestions for other behaviors are provided, such as going on a walk instead of snacking at home
8.3 Habit formation	Users are stimulated to schedule specific moments for their desired behavior
8.4 Habit reversal	n.a.
8.5 Overcorrection	n.a.
8.6 Generalization of target behavior	n.a.
8.7 Graded tasks	n.a.
**9. Comparison of outcomes**	
9.1 Credible source	Advice for healthy behavior is based on guidelines by national health institute
9.2 Pros and cons	Users are advised to write down the pros and cons of their past behavior and their desired behavior
9.3 Comparative imagining of future outcomes	Users are prompted to imagine in what way their life might improve when they change their behavior
**10 Reward and threat**	
10.1 Material incentive (behavior)	n.a.
10.2 Material reward (behavior)	n.a.
10.3 Non-specific reward	n.a.
10.4 Social reward	n.a.
10.5 Social incentive	n.a.
10.6 Non-specific incentive	n.a.
10.7 Self-incentive	MFB encourages users to reward themselves when they reach their goal, for example by going out with their partner of buying themselves a book.

10.8 Incentive (outcome)	n.a.
10.9 Self-reward	Users are advised to reward self of they have changed their behavior
10.10 Reward (outcome)	n.a.
10.11 Future punishment	n.a.
**11. Regulation**	
11.1 Pharmacological support	It is suggested to ask for pharmacological help with smoking when necessary
11.2 Reduce negative emotions	n.a.
11.3 Conserving mental resources	n.a.
11.4 Paradoxical instructions	n.a.
**12. Antecedents**	
12.1 Restructuring the physical environment	Advises to remove factors that might trigger unwanted behavior, for example ash trays or cookie jars
12.2 Restructuring the social environment	Advises to ask friends not to drink when meeting up
12.3 Avoidance/reducing exposure to cues for the behavior	Advises user to avoid locations that might have contextual cues for behavior such as pubs or candy stores.
12.4 Distraction	Suggests to go exercise when one craves a cigarette
12.5 Adding objects to the environment	Advises to place cues for desired behavior such as a fruit basket.
12.6 Body changes	Relaxation tips are provided to decrease stress and improve sleep
**13 Identity**	n.a.
**14 Scheduled consequences**	n.a.
**15 Self-believe**	n.a.
**16 Covert learning**	n.a.
