# Supplementary material for: A Web-Based Lifestyle Intervention Aimed at Improving Cognition in Patients With Cancer Returning to Work in an Outpatient Setting: Protocol for a Randomized Controlled Trial
Source: JMIR Res Protoc. 2021 Apr 26;10(4):e22670. doi: 10.2196/22670 (PMC8111506; doi:10.2196/22670)
Supplement: Multimedia Appendix 5 [file resprot_v10i4e22670_app5.docx]

| **Topic** | **Goal** |
| --- | --- |
| 1. Intervention | What does he/she think of the intervention, on what topics does he/she like to receive information and advice? |
| 2. Lifestyle domains | What does he/she think of the lifestyle domains covered?  Was the information in the intervention new or familiar to him/her? |
| 3. Information cognition and lifestyle | Does he/she want (more) specific information on cognitive impairment associated with cancer(treatment)? |
| 4. Behavioral change | Does he/she has experience with lifestyle changes (e.g. for weight/general health)  Did he/she experience any changes in thoughts and beliefs about lifestyle?  Did he/she change his/her lifestyle during the intervention?  What would be necessary to change his/her lifestyle? |
| 6. Facilitators | What would be facilitators to use an online lifestyle intervention?  What would motivate him/her to use an online lifestyle intervention? (both personal factors and program characteristics) |
| 7. Barriers | What would be barriers to use an online lifestyle intervention? (both personal factors and program characteristics) |
| 8. Missing features | What information was missing in the intervention? Does he/she has suggestions to improve the intervention? |

# Topic list for the semi-structured interviews for the feasibility study
