# Supplementary material for: A Web-Based Lifestyle Intervention Aimed at Improving Cognition in Patients With Cancer Returning to Work in an Outpatient Setting: Protocol for a Randomized Controlled Trial
Source: JMIR Res Protoc. 2021 Apr 26;10(4):e22670. doi: 10.2196/22670 (PMC8111506; doi:10.2196/22670)
Supplement: Multimedia Appendix 6 [file resprot_v10i4e22670_app6.docx]

The alcohol consumption questionnaire consists of 4 items. It assesses how many days a week alcohol is consumed, how many units are consumed daily on average and on days that the most alcohol is consumed. Finally, the amount of alcohol consumed weekly is reported.

The physical activity and exercise questionnaire consists of 5 items. It assesses how many times a week the patient engages in moderate physical activity and intense exercise, whether the patient knows the Dutch norm for physical activity, and how much physical activity the patients feels is needed to improve his/her physical health. Finally the participant is asked to grade his/her fitness with a number between 1 and 10.

The nutritional behaviour questionnaire consists of 7 items. The items investigate how many days a week a patient has breakfast, how many days a week the patient consumes 3 meals, how many days the patient consumes at least 2 pieces of fruit, how many days a week the patient eats at least 200 grams of vegetables, how many days a week the patient eats fish, how many days a week the patient eats after dinner, and how many days a week the patient eats unhealthy snacks between meals.

For non-smokers the smoking questionnaire consists of 1 item, and for smokers it consists of 4 items. The first item asks if the patient is a smoker. For smokers the next 3 questions will cover how many times the patient has tried to quit smoking, if the patient is considering to quit smoking, and how much the patient smokes on a daily basis.

The sleep questionnaire consists of 8 items. Subjects that are investigated are how many hours the patient sleeps, how much sleep the patient thinks he/she needs, at what time the patient goes to bed, at what time the patient wakes up, how long it takes to fall asleep, at what time the patient would prefer to go to bed, at what time the patient would prefer to wake up, and how many times a night the patient wakes up. The patient is also presented with 12 statements related to sleep and has to indicate whether he/she experienced the situation in the statement in the past 4 weeks.

The stress questionnaire is the Dutch translation of the International Stress Management Association’s stress questionnaire [1]. The questionnaire consists of 25 statements related to stress behaviour, where the participants have to indicate whether the statement is applicable to them.

1. Association, I.S.M. *International Stress Management Association (ISMA)-UK stress questionnaire*. 2013 [cited 2019 23-10-2019]; Available from: <http://www.isma.org.uk/wp-content/uploads/2013/08/Stress-Questionnaire.pdf>.
